# Antibacterial Activity of Pediocin and Pediocin-Producing Bacteria Against *Listeria monocytogenes* in Meat Products

**DOI:** 10.3389/fmicb.2021.709959

**Published:** 2021-09-17

**Authors:** Nasim Khorshidian, Elham Khanniri, Mehrdad Mohammadi, Amir M. Mortazavian, Mojtaba Yousefi

**Affiliations:** ^1^Department of Food Technology Research, National Nutrition and Food Technology Research Institute, Shahid Beheshti University of Medical Sciences, Tehran, Iran; ^2^Food Safety Research Center, Shahid Beheshti University of Medical Sciences, Tehran, Iran; ^3^Food Safety Research Center (Salt), Semnan University of Medical Sciences, Semnan, Iran

**Keywords:** bacteriocin, pediocin, antimicrobial, *Listeria monocytogenes*, meat

## Abstract

One of the most important challenges in the food industry is to produce healthy and safe food products, and this could be achieved through various processes as well as the use of different additives, especially chemical preservatives. However, consumer awareness and concern about chemical preservatives have led researchers to focus on the use of natural antimicrobial compounds such as bacteriocins. Pediocins, which belong to subclass IIa of bacteriocin characterized as small unmodified peptides with a low molecular weight (2.7–17 kDa), are produced by some of the *Pediococcus* bacteria. Pediocin and pediocin-like bacteriocins exert a broad spectrum of antimicrobial activity against Gram-positive bacteria, especially against pathogenic bacteria, such as *Listeria monocytogenes* through formation of pores in the cytoplasmic membrane and cell membrane dysfunction. Pediocins are sensitive to most protease enzymes such as papain, pepsin, and trypsin; however, they keep their antimicrobial activity during heat treatment, at low temperatures even at −80°C, and after treatment with lipase, lysozyme, phospholipase C, DNase, or RNase. Due to the anti-listeria activity of pediocin on the one hand and the potential health hazards associated with consumption of meat products on the other hand, this review aimed to investigate the possible application of pediocin in preservation of meat and meat products against *L. monocytogenes*.

## Introduction

Meat and meat products have an important role in the human diet, and their consumption has increased among animal food consumers as an excellent source of protein and other nutrients in recent years (Bohrer, 2017; Yousefi et al., 2018). These products have high nutritional value, but are highly perishable. In fact, meat and meat products are a suitable medium for growth of various pathogenic and spoilage microorganisms due to the presence of essential nutrients, absence of competing microorganisms, and desirable water activity and pH (Galvez et al., 2008). In addition, environments of food production and ingredients applied in the recipes of products such as frankfurters and sausages can facilitate microbial proliferation (Mamber et al., 2020). Therefore, meat and meat products must be produced and stored under safe and hygienic conditions. However, microbial contamination of meat and meat products is likely to occur in some poor hygienic conditions of processing and storage of these products, which can lead to safety and spoilage problems (Kurpas et al., 2018; Yousefi et al., 2020).

Contamination of meat products with pathogenic microbes is one of the main health and economic concerns all over the world, since serious foodborne diseases can be caused by their consumption and production and healthcare costs increase for food manufacturers owing to loss of productivity (Abatcha, 2017; Yousefi et al., 2020). Various pathogens such as *Listeria monocytogenes*, *Escherichia coli*, O157:H7, *Campylobacter jejuni*, *Salmonella* spp., and *Clostridium* spp. participate in incidence of foodborne diseases through the consumption of meat and meat products that were produced and stored under inappropriate conditions (Laranjo et al., 2019). Among these, *L. monocytogenes* is the causative agent of listeriosis, which is known as a major virulent foodborne disease (Cherifi et al., 2020; Pilevar et al., 2020a; Yousefi et al., 2020). It has been indicated that 172,823 disability-adjusted life-years (DALYs), 23,150 illnesses, and 5,463 deaths occurred in 2010 worldwide as a consequence of listeriosis (De Noordhout et al., 2014). Also, it is estimated that approximately 1,600 cases of listeriosis occur each year in the United States, and nearly 2,500 cases of listeriosis have been reported in European Union (EU) countries with a high mortality rate of about 20% in endangered population (EFSA, 2019; Ceruso et al., 2020). Therefore, the presence of *L. monocytogenes* in foodstuff and incidence of listeriosis is still considered as one of the most important food safety challenges worldwide (Ceruso et al., 2020).

Due to the ability of *L. monocytogenes* to grow in harsh conditions of processing or storage including low water activity, high concentrations of salt, pH ranges of 4.1–9.6, and refrigeration temperatures, it is difficult to control *L. monocytogenes* by food preservation techniques, and since this microorganism is able to grow at low temperatures (2–4°C), there is a specific concern about the presence of *L. monocytogenes* in meat and meat products (Shukla et al., 2017; Bucur et al., 2018; Chen et al., 2019; Fang et al., 2021). Various thermal and non-thermal preservation strategies have been applied to ensure the safety of food. Furthermore, different preservatives such as synthetic antimicrobial agents have been used to prevent microbial contamination through processing, distribution, and storage of food products such as meat products (Amit et al., 2017; López et al., 2019; Bahrami et al., 2020). Although synthetic additives used in the food industry are food-grade and Generally Recognized as Safe (GRAS), there is a growing concern about the use of these additives by consumers (Carocho et al., 2014; Khorshidian et al., 2018; Yousefi et al., 2020). Hence, there is an increasing attention in utilizing natural antimicrobial agents such as lactoperoxidase, lactoferrin, lysozyme from animal sources, essential oils and herbal extract from plant sources, and bacteriocin from microbial sources (Del Nobile et al., 2012; Hayashi et al., 2013; Carocho et al., 2015).

Various studies have carried out the use of various bacteriocins as food biopreservatives to inhibit pathogenic microbes (De Souza de Azevedo et al., 2019; Furlaneto-Maia et al., 2020; Xu et al., 2021). Nisin and pediocin are the most studied bacteriocin that could be utilized commercially as natural preservatives (Acuña et al., 2011). Bacteriocins are antibacterial peptides or proteinaceous toxins synthesized in ribosomes of bacteriocinogenic strains including *Lactococcus lactis*, *Pediococcus acidilactici*, and *Enterococcus faecalis* (Song et al., 2017; Balandin et al., 2019; Pilevar et al., 2020b; Yoon and Kang, 2020). These natural food biopreservatives kill or prevent the growth of a wide panel of Gram-positive foodborne pathogens, as well as spoilage bacteria (Pilevar et al., 2020b). Several studies have reported that bacteriocins produced by *L. sakei* and *L. curvatus* can diminish the number of *L. monocytogenes* in meat products (De Souza Barbosa et al., 2015; Casaburi et al., 2016; Castellano et al., 2018). It seems that they operate their antimicrobial activities by forming pores on target cell membranes, inhibiting the synthesis of nucleic acids, changing the electrostatic potential of microorganisms, and inhibiting the activity of certain enzymes (Basanta et al., 2009; Sirsat et al., 2009).

The use of effective bacteriocins against meat pathogens has gained attention in the food industry, especially for ready-to-eat (RTE) or fresh-tasting foods because their utilization can decline the application of intense thermal methods and chemical preservatives (Hernández-Aquino et al., 2019). On the other hand, at a daily intake of 2.9 mg/person, they are recognized safe for humans (Cleveland et al., 2001).

One of the bacteriocins is pediocin, which is a heat-stable peptide (Deegan et al., 2006; López et al., 2008) secreted by *Pediococcus* bacteria (Porto et al., 2017; Balandin et al., 2019). It is also able to tolerate low temperatures and retain its activity in a wide range of pH (Porto et al., 2017; Niamah, 2018). Numerous reports have stated that pediocin has an anti-listeria effect and can reduce the population of *L. monocytogenes* in various food products (Loessner et al., 2003; Bari et al., 2005).

Considering the anti-listeria activity of pediocin and pediocin-like bacteriocins on the one hand and the listerial resistance to nisin on the other hand, it seems that pediocin can be applied in meat and meat products alone or in combination with other preservation methods to hinder *L. monocytogenes* growth (Naghmouchi et al., 2006; Maciel et al., 2017; Castro et al., 2018). Therefore, we aim in this study to review the pediocin and pediocin-like bacteriocin, antimicrobial activity of these bacteriocins and their application against *L. monocytogenes* in meat and meat products.

## *Listeria Monocytogenes* and Its Presence in Meat Products

*Listeria* spp. are Gram-positive, facultative anaerobes, oxidase-negative, catalase-positive, and non-spore-forming bacteria (Doijad et al., 2018). *L. monocytogenes* is a member of the *Listeria* genus and a food-associated pathogen that is widely distributed in the nature (water, soil, and forage) and a broad range of foodstuffs (Shamloo Aghakhani et al., 2012; Jones and D’Orazio, 2013). Thirteen serotypes of it have been identified, and five of these serotypes (1.2a, 1.2b, 1/2c, 4c, and 4b) are most prevalent in food manufacturing plants or food (Meloni, 2015; Shamloo et al., 2019). Listeriosis is a human infection caused by consumption of contaminated foods with *L. monocytogenes* that can lead to severe symptoms such as spontaneous abortion in pregnant women, meningitis, and septicemia (Kim et al., 2018; Kurpas et al., 2018). Furthermore, it has been reported that foodborne listeriosis could present as a gastrointestinal illness with fever in non-immunocompromised patients (Aureli et al., 2000). It has been demonstrated that the large numbers of microbes (>10^3^ cfu/g) are needed for the severe form of listeriosis (Churchill et al., 2019). One of the highest mortality rates are attributed to the foodborne disease listeriosis, so *L. monocytogenes* infections are considered as a life-threatening illness for high-risk groups including elderly, immunocompromised, pregnant women, and newborns (Zhang et al., 2021).

*Listeria monocytogenes* is a potential microbiological risk for raw meat and meat products due to its unusual ability to adapt at cold temperatures, even at 1°C (Conficoni et al., 2016). Additionally, this bacterium can multiply to threatening levels in meat products at any step of the food chain because (a) meat products with pH value above 5 are an appropriate medium for the growth of this organism (Zhu et al., 2005), (b) *L. monocytogenes* can tolerate nitrite and salt up to 12% (Gandhi and Chikindas, 2007; Camargo et al., 2017), (c) modified atmospheres have no impact on its survival (Meloni, 2019), and (d) its capability to form biofilms on food contact surfaces or with other bacteria in meat production establishments increases its resistance to UV light, sanitizers, and bactericide agents (Doijad et al., 2015; Kurpas et al., 2018). The highest adhesion to the surfaces is attributed to serotypes 1/2a, 1/2b, and 4b (Meloni et al., 2014). Therefore, the possibility of cross-contamination and the growth of *L. monocytogenes* are enhanced to an unsafe level in finished products. Many researches have been published about the incidence of *L. monocytogenes* in foods of animal origin around the world. Mohamed et al. (2016) investigated 150 samples of processed meat for the presence of *L. monocytogenes* from October 2013 to September 2014 in Egypt. They found that 4% of minced meat, beef burger, and luncheon samples were infected with *L. monocytogenes*. In a survey conducted by Benhalima et al. (2019) over a 10-year period in China, RTE meats and raw meats were contaminated with *L. monocytogenes* 3.2 and 8.5%, respectively, and meat products from northeastern and central China had the highest occurrence of *L. monocytogenes*. In East Algeria, the prevalence of *L. monocytogenes* in sausage was 33.3% (Liu et al., 2020). In another research in Spain, the incidence of *L. monocytogenes* in RTE meat products between 2012 and 2013 was 17.14% in cooked products, 36.84% in raw-cured products, and 24.32% in dry-cured, salted products (Gómez et al., 2015). Also, D’Ostuni et al. (2016) isolated *L. monocytogenes* from 4.2% of raw pork sausages and 2.4% of entrails lamb rolls examined from 2008 to 2014 in Southern Italy. It appears that bacterial contamination of meat products can occur in processing lines and equipment and during postprocessing phases (slicing and packaging). With regard to the sensitivity of *L. monocytogenes* to the thermal process and its inactivation after cooking (Martín et al., 2014), the major concern is recontamination of processed food with *L. monocytogenes*, which affects their shelf life (Bakhtiary et al., 2016). Thus, from the viewpoint of food safety, the development of appropriate control strategies against this organism is an important issue in food industry.

## Bacteriocin

As mentioned before, production of safe food free from microorganism especially pathogens needs to considered as one of the most important priorities of the food industry. Due to the achievement to this important aim, various strategies have been examined, among which attention has been drawn to utilizing natural antimicrobial agents such as essential oils and bacteriocins (Carocho et al., 2015; Mokoena, 2017; Yousefi et al., 2020).

**FIGURE 1:**
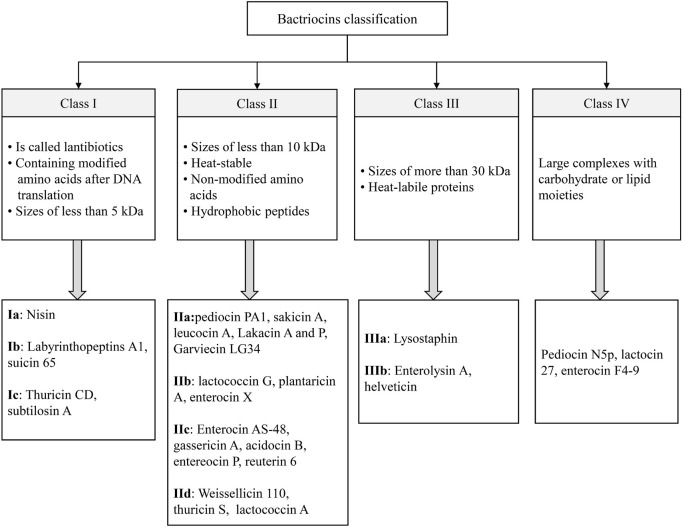
The main classes and sub-classes of bacteriocins.

Bacteriocins as natural antimicrobial agents are ribosomally synthesized peptides or proteins that are produced by G+ and G– bacteria such as lactic acid bacteria (LAB), *Staphylococcus* strains, *Bacillus* strains, and *E. coli* strains. Without having an adverse effect on the bacteria that produce them, bacteriocins have inhibitory effects on various groups of undesirable microorganisms, and their activities are mostly targeted at the cell wall of microorganisms (Espitia et al., 2016; Gutiérrez-Cortés et al., 2018; Lopetuso et al., 2019; Ng et al., 2020; Benítez-Chao et al., 2021). They are small cationic molecules made up of approximately 30–60 amino acids forming amphiphilic helices showing good stability at 100°C for 10 min. They are different in terms of genetic origin, biochemical properties, molecular weight (MW), and activity spectrum (Parada et al., 2007; Mokoena, 2017; Sidooski et al., 2019). The bacteria that are taxonomically close may be inhibited by narrow-spectrum bacteriocins, while a wide variety of bacteria are inhibited by broad-spectrum bacteriocins (Cotter et al., 2005; O’Connor et al., 2020). Based on the producer organism, MW, chemical structure, existence of modified amino acids, and thermal stability, the bacteriocins can be classified into four groups (Class I, II, III, and IV) (López-Cuellar et al., 2016; Johnson et al., 2018; Kumariya et al., 2019; Cui et al., 2021). The main classes and sub-classes of bacteriocin and their characteristics are shown in Figure 1. Nisin and pediocin are the most studied bacteriocin that could be utilized commercially as natural preservatives (Acuña et al., 2011). Nisin is currently the only bacteriocin that can be used as an authorized additive. However, pediocin as a food ingredient produced by *P. acidilactici*, a pediocin-producing strain, can be commercially exploited for food preservation, and its application is covered by several US and European patents (El-Ghaish et al., 2011; Espitia et al., 2016).

## Pediocin as Natural Antimicrobial Agent

Pediocin belongs to subclass IIa and is produced from *Pediococcus* spp. such as *P. acidilactici*, *P. claussenii*, *P. cellicola*, *P. damnosus*, *P. ethanolidurans*, *P. inopinatus*, *P. parvulus*, *P. pentosaceus*, and *P. stilesii* (Haakensen et al., 2009; Porto et al., 2017). The bacteriocins generated from these species are generally called pediocin. However, based on the isomer and producing strain, the symbols have been added with the pediocin word such as pediocin AcH, pediocin SJ-, pediocin JD, and pediocin PA-1 (Bhunia et al., 1991; Christensen and Hutkins, 1994; Rodríguez et al., 2002; Niamah, 2018).

Pediocins as natural bacteriocins are the biomolecules that exert a broad spectrum of antimicrobial activity against Gram-positive bacteria, especially against pathogenic bacteria, such as *L. monocytogenes*. Due to its activity against *L. monocytogenes*, it could be applied in various food products to control the growth of this food-borne pathogen that is a concern in the food industry (Mokoena, 2017; Porto et al., 2017). Pediocin can be utilized in the food industry through two approaches, either through the *in situ* method by adding *Pediococcus*, *Enterococcus*, or *Lactobacillus* strains to the food matrix to produce pediocin under controlled conditions to prevent the growth of pathogens in food, or direct use of the optimal concentration of pediocin to the food matrix (Silva et al., 2018; Ng et al., 2020).

## Properties and Structural Properties of Pediocin

As mentioned, Class II of bacteriocins is described as the compounds with sizes of less than 10 kDa, heat-stable, non-modified, and hydrophobic peptides. Among this, Class IIa (pediocin-like or *Listeria*-active) has shown to exert high specific activity against the food pathogen *L. monocytogenes* (Espitia et al., 2016).

Pediocins are characterized as small unmodified peptides that have a MW of lower than 5 kDa (Papagianni and Anastasiadou, 2009). Pediocin peptides are composed of 40–44 amino acids of both aliphatic and aromatic amino acid with no posttranslational modification. The amino acid similarity in the sequence of pediocin-like bacteriocins is about 40–60%, and this sequence of amino acid presents a conserved N-terminal hydrophobic region in the YGNGV motif and a variable C-terminal hydrophobic or amphiphilic region (Papagianni, 2003; Porto et al., 2017; Niamah, 2018). A significant reduction in anti-listeria activity occurred by the substitution of a single amino acid residue (Sun et al., 2015).

Pediocin is principally present in unstructured conformations as random coils in watery solutions, while, in non-aqueous solutions, it makes a partly helical structure with different amounts of hydrophobicity (Ennahar et al., 2000). The structure of pediocin basically consisted of two regions: a hydrophilic cationic region (N-terminal) and a hydrophobic/amphiphilic region (C-terminal) (Johnsen et al., 2005).

The N-terminal region shows the three-stranded antiparallel β-sheet supported by a disulfide bridge consisting of two cysteine residues (C9 and C14) (Drider et al., 2006; Porto et al., 2017). At the end of the structure, the hairpin domain is created by a C-terminal tail with two cysteine residues that fold back onto the central α-helix by a disulfide bridge. Furthermore, a flexible hinge that presents among the N-terminal region and the hairpin domain in the C-terminal region makes two of these regions to move relative to each other (Espitia et al., 2016).

The known pediocins produced from *P. acidilactici*, *P. damnosus*, and *P. pentosaceus* strains are heat-resistant small-structure hydrophobic peptides. Their bactericidal activities can be maintained during heat treatment, sometimes even at sterilization temperatures. Furthermore, pediocins are able to tolerate low temperatures even at −80°C (Anastasiadou et al., 2008b; Papagianni and Anastasiadou, 2009; Porto et al., 2017; Niamah, 2018; Ghosh et al., 2019). They also maintain their activity after treatments with lipase, lysozyme, phospholipase C, DNase, or RNase, while they are sensitive to most protease enzymes such as papain, pepsin, and trypsin (Wu et al., 2004; Anastasiadou et al., 2008a, b; Papagianni and Anastasiadou, 2009; Espitia et al., 2016; Niamah, 2018). Pediocin activity is retained in a wide range of pH. The isoelectric point of pediocin is 8.6–10, and it has a positive charge between (+3) and (+7) in pH 6 (Venema et al., 1997; Porto et al., 2017; Niamah, 2018). It has been reported by Papagianni and Anastasiadou (2009) that most isoforms of pediocin are thermally stable and remain active in a wide range of pH (2–10). It has been indicated that the main difference between the pediocin isoforms is related to their sensitivity against protease enzymes such as chymotrypsin, papain, pepsin, pronase E, proteinase K, and trypsin (Porto et al., 2017).

The effect of various treatments on antimicrobial activity of pediocin isoform is summarized in Supplementary Table 1.

## Antimicrobial Activity of Pediocin and Mode of Action

Antimicrobial activities of bacteriocins are carried out through various mechanisms such as destruction of cell wall and membrane integrity, interference with cell wall formation, inhibition synthesis of protein, and inhibition of gene expression. These bactericidal mechanisms depend on the class of bacteriocins and indicator bacteria (Balciunas et al., 2013; Cavera et al., 2015; Porto et al., 2017; Cui et al., 2021).

**FIGURE 2:**
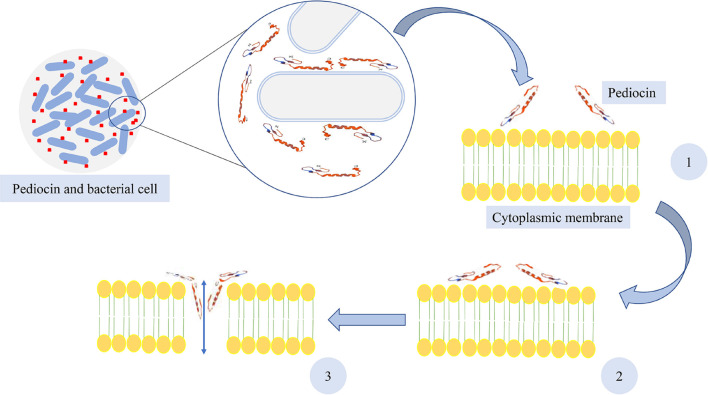
Pore formation of pediocin in bacterial cell membrane. (1) Adsorption of pediocin, (2) distribution and interaction of pediocin on the surface of the cytoplasmic membrane, and (3) pore formation in the membrane.

Pediocin, like other Class II bacteriocins, participates in creating partial or total imbalance of transmembrane proton distribution in sensitive cells (Bruno and Montville, 1993; Cleveland et al., 2001; Papagianni, 2003). In fact, the cytoplasmic membrane of bacteria is the pediocin’s target. All pediocin variants are known for their inhibitory activities against Gram-positive bacteria, especially *L. monocytogenes*. However, it has been indicated that pediocin S and L from *P. pentosaceus* S and L were also effective against Gram-negative microorganisms (Yin et al., 2003). The antimicrobial activity of pediocin depends on its structure and is carried out by formation of pores in the target membrane. These pores lead to leakage of ions and other cell compounds, release of cytoplasmic adenosine triphosphate (ATP), and inhibition of proton motive force (PMF) for energy production, and finally, when the leakages are more than the limit, cell death occurs (Montville and Chen, 1998). An initial connection between the target bacteria and pediocin PA-1 occurs before a pore is made in the bacterial membrane. This initial connection is principally occurred through electrostatic interactions created by the cationic and anti-parallel region of the β-sheet in the N-terminal region of pediocin with the lipoteichoic acid, the main component on the surface of Gram-positive bacteria (Espitia et al., 2016). The native divalent cations from cell surface is removed by this electrostatic interactions and makes the outer membrane unstable. Therefore, the entrance of the peptide and further peptide contact with the cytoplasmic membrane is facilitated (Powers and Hancock, 2003; Espitia et al., 2016). It has been indicated by Nissen-Meyer and Nes (1997) that the lipid composition of the target cell membrane is probably a substantial factor in the sensitivity of the bacteria to the pediocin PA-1 and other bacteriocins. It has also been demonstrated by Chen et al. (1997) that the affinity between phospholipid vesicles and pediocin PA-1 was enhanced by the presence of anionic lipids. After contacting and distribution of pediocin on the surface of the bacterial cytoplasmic membrane, the pores are formed by insertion of the hydrophobic, C-terminal, hairpin-like domain into the membrane (Johnsen et al., 2005; Drider et al., 2006). Furthermore, structural flexibility that is provided by the hinge makes the C-terminal, hairpin domain penetrate the hydrophobic part of the membrane (Espitia et al., 2016). It has been indicated that salts and amino acid cellular efflux were affected by pediocin PA-1 and the transmembrane electrical potential is also dissipated by this bacteriocin (Chikindas et al., 1993). Furthermore, it has been demonstrated by Bauer et al. (2005) that K+ loss occurred as a consequence of purified PD-1 pediocins and formation of pores on the cytoplasmic membrane in *Oenococcus oeni* cells. They also found that K+ loss is dependent on pH, and when reduced from 7 to 5, membrane disruption and K+ loss increased. Additionally, it has been stated that beside pore formation and cell membrane dysfunction, pediocin molecules enter the cell and create complexes between pediocin and cell components such as DNA, proteins, and enzymes (Montville and Chen, 1998). The antimicrobial activity of pediocin and formation of pores in the cytoplasmic membrane of bacterial cell are illustrated in Figure 2.

## Anti-Listeria Activity of Pediocin and Pediocin-Producing Bacteria in Meat Products

Generally, bacteriocins such as pediocins can be applied in food preservation through two methods: (a) inoculation of the bacteriocin-producing strain into the food matrix under desirable conditions to produce antimicrobial peptide *in situ* and (b) direct addition of bacteriocins to the food matrix (Perez Espitia et al., 2012). The first strategy and the inoculation of pediocin-producing strains into the food matrix can be considered as an effective alternative method to preserve meat products, due to pediocin’s ability to inhibit *L. monocytogenes*, *Clostridium perfringens*, and *Clostridium botulinum* (Okereke and Montville, 1991; Foegeding et al., 1992; Nieto-Lozano et al., 2006, 2010; Bagenda et al., 2008). However, it is necessary to be careful in selecting pediocin-producing strains based on the type of food in order to produce pediocin in adequate amount. In another method, commercially available pediocin that is produced at a laboratory or industrial scale is directly added into food products (Espitia et al., 2016). However, some limitations such as degradation by proteolytic enzymes, adsorption to food components, and variation in the solubility of pediocin should be taken into consideration when it is directly utilized in food products (Coma, 2008). In this section, the anti-listeria activities of direct addition of pediocin as well as pediocin-producing bacteria in the meat and meat product are reviewed.

In a study that is carried out by Nielsen et al. (1990), the use of bacteriocin produced by *P. acidilactici* was evaluated to inhibit *L. monocytogenes* in fresh meat. They figured out that the pediocin-treated meat showed 1–2.5 log cycles fewer in attachment of the *L. monocytogenes* than the control. Furthermore, their results revealed that after 2-min exposure with pediocin, a decrease of 0.5–2.2 log cfu/ml occurred in the population of attached bacteria depending on bacteriocin concentration. They also reported that the tested bacteriocin was able to exert an inhibitory effect against *L. monocytogenes* after 28 days at 5°C, and its residual activity was detected on the meat surface for at least 28 days at refrigerated storage.

It has been demonstrated by Berry et al. (1991) that the ability of bacteriocin-producing *P. acidilactici* JD1–23 to control *L. monocytogenes* contamination of frankfurters was dependent on the concentration of *Pediococcus*, the atmosphere, and the temperature of packaging. They reported that coinoculation of high levels (10^7^ cfu/g) of *P. acidilactici* JD1–23 and *L. monocytogenes* in frankfurters under vacuum packaging at 4°C inhibited the growth of pathogen up to 60 days. Similarly, Motlagh et al. (1992) studied the effect of pediocin AcH in controlling tree strains of *Listeria* in various food products and found that the bacteriocin action was dependent on concentration and strain. They reported a decrease of 1, 3, and 7 log cfu/g occurred in the population of *L. monocytogenes* ScottA, *L. monocytogenes* Ohio2, and *L. ivanovii* ATCC 19119, respectively, as a consequence of using 1,350 AU/ml of pediocin. They also indicated that this activity was immediate and independent of food types.

The inhibition of *L. monocytogenes* using the pediocin-producing strain *P. acidilactici* JD1–23 as starter culture during the manufacture of fermented semidry sausage was studied by Berry et al. (1990). They found that a 2 log cfu/g reduction of *L. monocytogenes* occurred during fermentation, while less than 1 log cfu/g decrease was observed in the *L. monocytogenes* population in the sausage fermented with a non-inhibitory *Pediococcus* strain. They also stated that *L. monocytogenes* was also inhibited in the sausage with pH more than 5.5, indicating that bacteriocin is produced independently of carbohydrate fermentation.

Furthermore, *in situ* production of pediocin using *P. acidilactici* during dry fermentation of sausage in order to control *L. monocytogenes* was studied by Foegeding et al. (1992). They understood that the *L. monocytogenes* population was reduced over dry sausage fermentation process, and an effective inactivation of *L. monocytogenes* was obtained when an adequate pH drop (below 4.9) occurred at the end of the fermentation process. Furthermore, they indicated that when pH was not lowered sufficiently during the fermentation, bacteriocin production also facilitated the reduction of any remaining *L. monocytogenes*. They also recommended that it is useful to apply bacteriocin-producing starter cultures to enhance control of *L. monocytogenes* in meat fermentations.

The effect of *P. acidilactici* H or Pediocin AcH on the behavior of *L. monocytogenes* strains in wiener sausage exudates was evaluated by Yousef et al. (1991). They recognized that both methods were effective in decreasing the growth of pathogens during storage of wiener sausage exudates at 4 or 25°C. They also mention that, although rapid initial reduction occurred in a numbers of pathogens, using the producing strain resulted in the lower final levels. This reduction in counts might be due to the production of pediocin AcH during late logarithmic growth.

Similarly, the anti-listeria ability of *P. acidilactici* JBL1095 (pediocin AcH producer) and a non-bacteriocin producer (*P. acidilactici* LB42) in the vacuum-packaged wieners was evaluated by Degnan et al. (1992). The results showed that there were no significant changes in the *L. monocytogenes* or *P. acidilactici* population in the treated and untreated samples during 72 days of storage at 4°C. On the other hand, during 8 days of storage at 25°C, a slight (0.33 log10 cfu/g) and significant (2.7 log cfu/g) decrease in the counts of *L. monocytogenes* occurred in packages containing strain LB42 and JBL1095 held at 25°C, respectively. They stated that the bacteriocin producer was not able to grow or produce the bacteriocin at 4°C, while a remarkable anti-listeria effect was observed at 25°C due to production of bacteriocin.

Additionally, genomic analysis and anti-listeria activity of three pediocin-producing (Ped+) and two non-pediocin-producing (Ped–) strains of *P. acidilactici* in the preparation of turkey summer sausage were studied by Luchansky et al. (1992). The results showed that equivalent amounts of acid were produced by all the starter culture acid during fermentation. However, the present of Ped+ starter culture resulted in greater reduction of *L. monocytogenes* (3.4 log cfu/g) in comparison to the samples containing Ped- starter culture (0.9 log cfu/g) and remarkable pediocin activity was determined from sausages prepared with the Ped+ strain during at least 60 days storage at 4°C. They claimed that all the commercially available starter cultures are not equal in encoding pediocins or as effective as each other in controlling *L. monocytogenes*. Therefore, selection of an effective strain should be taken into consideration.

The anti-listeria activity of pediocin (3,000 AU/ml) in the slurries of beef muscle tissue and beef tallow that were contaminated with 2.5 × 10^5^ cfu/ml of two *L. monocytogenes* strains was studied by Degnan et al. (1993). The greatest reduction in *Listeria* counts was carried out within 1.5 min of pediocin addition, and after that, the population of *Listeria* did not change; however, the activity of pediocin continued to decrease in the treatment for up to 60 min. They noted that this reduction might be driven by the cumulative effects of proteolysis and association with both protein and lipid. They also indicated that encapsulation of pediocin within phosphatidyl-choline-based liposomes before addition to the slurries resulted in higher activity of pediocin in comparison to free pediocin (Degnan et al., 1993). Furthermore, it has been claimed that cold storage of *L. monocytogenes*-contaminated ground pork (10^3^ cfu/g) in the presence of pediocin (8,192 AU/g) led to a 2 log cfu/ml decrease in the population of *L. monocytogenes* in comparison to the sample free from pediocin, regardless of whether the samples were stored in air, vacuum, or modified atmosphere (Khojasteh and Murano, 1996).

The anti-listeria activity of pediocin AcH bound to heat-killed *P. acidilactici* cells in irradiation-sterilized raw chicken breast meat that was contaminated with *L. monocytogenes* Scott was studied by Goff et al. (1996). They figured out that pediocin-treated samples showed anti-listeria activity both before and after cooking. They claimed that this might be useful in protecting the consumers from bacterial post-processing recontaminations and/or undercooking. Similarly, Mattila et al. (2003) bound pediocin AcH to heat-killed producer cells of *Lactobacillus plantarum* WHE 92 by adjusting the pH of the medium to 6.0, and the preparation was added on the sliced cooked sausage that inoculated with *L. monocytogenes* ATCC 7644 (2.7 log cfu/g). Their results showed that there were no significant differences among the treated and control samples in terms of pH value, flavor, and growth of LAB. However, in the pediocin-treated sample, *L. monocytogenes* population was decreased to <2 log cfu/g after 6 days of storage and no more reduction occurred through remaining storage time, while in the control sausage, the counts of *L. monocytogenes* remained at the inoculated level. They indicated that due to the presence of *Listeria* in the treated sample at the end of storage, pediocin was not efficient enough to kill all *L. monocytogenes*. Therefore, it seems that this bacteriocin should be applied in hurdle technology to improve the efficiency (Mattila et al., 2003).

Furthermore, Chen et al. (2004c), investigated the ability of pediocin (ALTA 2341) in controlling *L. monocytogenes* on frankfurters. In this regard, the surface of frankfurters was inoculated with a five-strain mixture of *L. monocytogenes* (3.40 or 5.20 log cfu/g) and treated with 3,000 or 6,000 AU of pediocin per link. The treated samples were vacuum-packaged (as 1, 5, or 10 links per package) and stored at different temperatures (4, 10, and 25°C) for 12 weeks. The results revealed that the extent of *Listeria* inhibition was highly temperature-dependent and the most effective reduction occurred in the pediocin-treated frankfurters. They stated that the frankfurters treated with 6,000 AU of pediocin and stored at 4°C prevented the growth of *L. monocytogenes* for at least 7 weeks and reduced the growth of this pathogen for up to 12 weeks. They claimed that in order to achieve sufficient inhibition of *Listeria*, pediocin should be applied in combination with well-controlled temperature or other complementary inhibitory methods (Chen et al., 2004c).

Nieto-Lozano et al. (2006) studied the anti-listeria effect of bacteriocin, produced by *P. acidilactici* on Spanish raw meat. The pediocin-treated meat samples (500, 1,000, or 5,000 bacteriocin U/ml) were contaminated with *L. monocytogenes* and stored at 15°C for 72 h or at 4°C for 21 days. They figured out that application of 500, 1,000, or 5,000 pediocin U/ml (BU/ml) led to a 1, 2, or 3 log cfu/g decrease in the population of *L. monocytogenes* after 72 h storage at 15°C, respectively, indicating that this reduction was dependent on the concentration of pediocin. They also reported that the treatment with 1,000 or 5,000 BU/ml decreased the counts of *L. monocytogenes* by 2.5 and 3.5 cfu/ml after 21 days storage at 4°C, respectively, compared to the control (Nieto-Lozano et al., 2006). Similarly, Nielsen et al. (1990) demonstrated that reduction in *L. monocytogenes* population was dependent on the inoculum of *L. monocytogenes* and the pediocin PA-1 concentration.

In the other study, which was carried out by Nieto-Lozano et al. (2010), anti-listeria activities of pediocin PA-1 and the *P. acidilactici* MCH14 pediocin-producing strain were investigated in frankfurters and Spanish dry-fermented sausages. Their results showed a 2 log cycles decrease in treated Spanish dry-fermented sausages in comparison to the control sample after 30 days storage (Nieto-Lozano et al., 2010). It has been suggested that the bactericidal effect of the pediocin-producing strain of *P. acidilactici* was due to bacterial lysis (Heo et al., 2007). It has also been reported that application of 5,000 BU/ml pediocin PA-1 led to a 2 and 0.6 log cycle decrease in *Listeria* counts in the frankfurters stored at 4°C for 60 days and at 15°C for 30 days, respectively, when compared to the control samples (Nieto-Lozano et al., 2010). Their results also revealed the importance of storage temperature in the effectiveness of bacteriocin such as pediocin as stated in previous investigations (Degnan and Luchansky, 1992; Nieto-Lozano et al., 2006). Therefore, due to the greater inhibitory effect of pediocin PA-1, this bacteriocin and related pediocin-producing strain could be applied in refrigerated products (Nieto-Lozano et al., 2010).

Furthermore, the ability of bacteriocin-producing *P. acidilactici* 13 and its antimicrobial substance against *L. monocytogenes* during ripening of dry fermented sausage (sucuk) and storage of sliced turkey breast was investigated by Cosansu et al. (2010). *P. acidilactici* 13 was isolated from naturally fermented sucuk, and its application as a starter culture for sucuk production resulted in a 3.32 log cfu/g decrease in population of the pathogen during 8 days of storage. On the other hand, in the control sample, 1.37 log cfu/g occurred, while a purified antimicrobial substance (6,400 AU/ml) produced by *P. acidilactici* 13 led to an instant reduction of *L. monocytogenes* (1.03 log cfu/cm^–2^) in turkey breast slices stored at 12°C for 10 days. However, application of partially purified antimicrobial substance was not able to inhibit growth of *L. monocytogenes* for 10 days of storage at 12°C. Therefore, they proposed that this partially purified substance of *P. acidilactici* 13 could be utilized in combination with other preventative strategies against *L. monocytogenes* in meat products that do not undergo a fermentation step (Cosansu et al., 2010).

Kingcha et al. (2012) found that inoculation of *P. pentosaceus* BCC 3772 in Nham, a Thai traditional fermented pork sausage, resulted in a significant reduction in the growth of *L. monocytogenes* ATCC 19115 over 18–24 h of fermentation without any significant changes in sensory properties of the final fermented Nham products. They also observed a correlation among *P. pentosaceus* BCC3772 inoculum and anti-listeria effect, indicating the importance of pediocin concentration in the prevention the growth of *Listeria*. It seems that the differences in anti-listeria activity of various concentrations of pediocin or the loss of antimicrobial activity of pediocin, especially during long times, could be attributed to the binding of the pediocin to the food matrix and proteolytic degradation by enzyme. In this regard, Kingcha et al. (2012) pointed out that inoculation of 10^4^ cfu/g *P. pentosaceus* BCC 3772 in Nham led to a rapid reduction of *L. monocytogenes* counts to <2 log cfu/g over 18–24 h of fermentation. However, a slight increase in the population of *L. monocytogenes* was observed after 36 h of fermentation, indicating the possible loss of pediocin.

**FIGURE 3:**
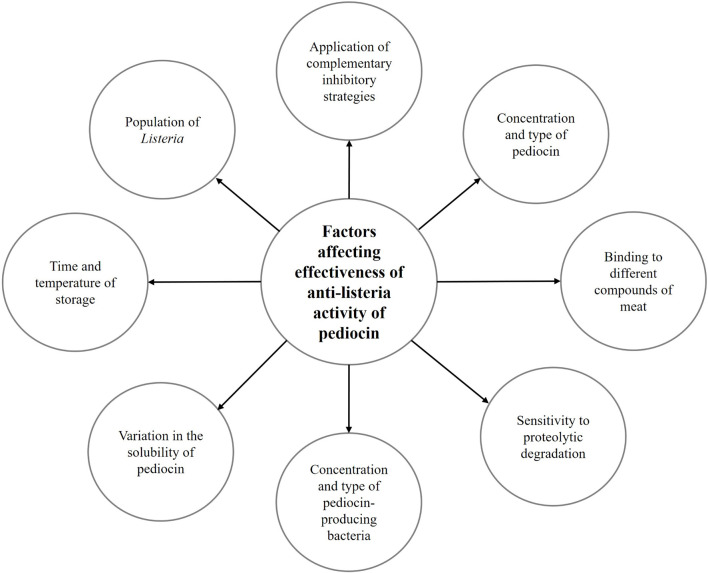
Factors affecting the effectiveness of pediocin in inhibition of *Listeria* in meat and meat products.

Furthermore, Kiran and Osmanagaoglu (2014) studied the anti-listeria activity of purified pediocin AcH/PA-1, produced by *P. pentosaceus* OZF in chicken meat products that were radiated and inoculated with 10^5^ cfu/g of *L. monocytogenes*. They reported that there was significant reduction in the *Listeria* counts (3.8 log cfu/g) in the pediocin-treated sample in comparison to control after 14 days of storage at 4°C. On the other hand, re-growth of *L. monocytogenes* was observed after more storage time (3–4 weeks), indicating the possible degradation of the tested pediocin by proteases derived from raw meat products or its interaction with different compounds of meat. They suggested that more effective protection of bacteriocin such as pediocin can be achieved through multi-hurdle preservation methods (Kiran and Osmanagaoglu, 2014). Figure 3 shows the factors that affect the efficacy of anti-listeria activity of pediocin in meat and meat products.

As mentioned before, various studies have been using pediocin, the bacteriocin-like inhibitory substance of *Pediococcus* strain, and pediocin-producing bacteria in various meat products and explored the anti-listeria activity of these components (Mattila et al., 2003; Cosansu et al., 2010; Nieto-Lozano et al., 2010; De Azevedo et al., 2020). However, it has been noted that due to the sensitivity of these compounds to proteolytic degradation, binding to food compounds and variation in the solubility of this bacteriocin have limited the direct application of pediocin, especially in low concentration or during long-term storage at improper temperatures. Therefore, in addition to these applications, pediocin has shown potential for use as a part of hurdle technology along with other preventative strategies or as part of antimicrobial agents incorporated into packaging materials.

As aforementioned, Chen et al. (2004c) investigated the ability of pediocin (ALTA 2341) in controlling *L. monocytogenes* on frankfurters and presented that in order to achieve sufficient inhibition of *Listeria*, pediocin should be applied in combination with well-controlled temperature or other complementary inhibitory methods (Chen et al., 2004c). Therefore, in the next investigation carried out with same authors, irradiation was applied in combination with pediocin (ALTA 2341) to control the growth of *L. monocytogenes* on frankfurters. Similar to a previous study, the artificially contaminated frankfurters were treated with 3,000 or 6,000 AU of pediocin per link and vacuum packaged as 1, 5, or 10 links per package. After packaging, 1.2 or 2.3 kGy irradiation dose was applied for 1-link and 5-link packages, while the 10-link packages were irradiated at 1.4 or 3.5 kGy. The treated and untreated samples were stored at 4, 10, and 25°C for 12 weeks. The results showed that in order to reach a 50% reduction of *L. monocytogenes* on frankfurters in 1-link or 5-link packages, pediocin should be applied with postpackaging irradiation at 1.2 kGy or more. They demonstrated that application of 6,000 AU pediocin in combination with 2.3 kGy or higher irradiation dose was effective in preventing the pathogen in all package sizes stored at 4 and 10°C for 12 weeks. They claimed that application of pediocin did not have an adverse effect on the sensory properties of frankfurters, and these synergistic effects between pediocin and irradiation in combination with cold storage (4°C) led to little or no growth of the *L. monocytogenes* in the 1-link or 5-link packages during 12 weeks of storage (Chen et al., 2004b). In another similar study performed by the same authors, instead of irradiation, postpackaging thermal pasteurization was exerted in combination with pediocin (ALTA 2341) to inhibit *L. monocytogenes* on frankfurters. For this purpose, the vacuum-packed frankfurters were heated in hot water at 71, 81, and 96°C for 30, 60, and 120 s, respectively. They figured out that the anti-listeria effect of thermal pasteurization was dependent on package size, and the most and least effective heat treatment for pediocin-treated samples were observed in 1-link and 10-link packages, respectively. Their results showed that a 50% reduction of initial inoculations was obtained in the treatment with 6,000 AU/g pediocin utilized in combination with heat treatment of 81°C or more for at least 1 min. They also indicated that little or no growth of *L. monocytogenes* was observed on the surface of frankfurters stored at 4 or 10°C for 12 weeks and at 25°C for 12 days. They concluded that application of pediocin along with postpackaging thermal treatment can be considered as an efficient treatment to enhance control of *L. monocytogenes* on frankfurters (Chen et al., 2004a). Furthermore, the combined effect of food additives and pediocin in inhibition of *L. monocytogenes* has shown that the anti-listeria activity of food additives increased by combination use with pediocin. For example, the anti-listeria effects of sodium diacetate in combination with pediocin (5,000 AU/ml) in turkey slurries were studied by Schlyter et al. (1993). Their results revealed a listericidal effect (ca. 7 log cfu/ml) in treatments containing pediocin with 0.5% diacetate at 25°C and pediocin with 0.3% diacetate at 4°C. They stated that the increased anti-listeria activity of diacetate in combination with pediocin was due to synergistic effects. They concluded that it is recommended to utilize multiple barriers such as diacetate in combination with pediocin for increased control against *L. monocytogenes* in turkey. Additionally, Maks et al. (2010) studied the interaction effect of temperature (56.3–60°C) and pediocin (0–10,000 AU) on the thermal inactivation of *L. monocytogenes* on bologna. They figured out that by increasing the concentration of pediocin from 0 to 5,000 AU, *D*-values decreased, while a further increase (to 7,500 and 10,000 AU) has a protective effect on thermal inactivation. They also mentioned that application of 10,000 AU pediocin in combination with sodium lactate or sodium diacetate at 56.3 and 60°C, respectively, led to a slight increase in predicted *D*-values. Their results revealed that the interaction effects between additives could be different at various temperatures/concentrations and therefore food manufacturers should carefully modify food formulations and evaluate them with sufficient tests to ensure that the safety of the product is not compromised. Similarly, Grosulescu et al. (2011) developed a predictive model in order to interpret the effect and interaction of sodium diacetate (0–2.5%), sodium lactate (0–4.8%), and pediocin (0–10,000) on thermal resistance (56.3–60°C) of starved *L. monocytogenes* on bologna. They found that addition of pediocin (up to 5,000 AU) by increasing temperature and sodium diacetate decreased *D*-values. On the other hand, a slight increase in the *D*-value occurred when pediocin was utilized in concentrations of 7,500 or 10,000 AU (Grosulescu et al., 2011). In accordance with these studies, Gupta (2005) found that the addition of pediocin up to a concentration of 7,500 AU decreased thermal resistance of *L. monocytogenes*, whereas further addition of pediocin (10,000 AU) led to a slight increase in heat resistance of *L. monocytogenes*.

In the other hurdle technology, mild high hydrostatic pressure (HHP) (300 MPa, 10°C, 5 min) and *P. acidilactici* HA-6111-2 or its bacteriocin, pediocin PA-1 (1,280 AU/g), was utilized as a potential hurdle technology to control *L. monocytogenes* in Portuguese traditional fermented meat sausages. They understood that *L. monocytogenes* was undetectable at 14 and 21 days of refrigerated storage in the samples treated only with PA-1 or *P. acidilactici* HA-6112, respectively. However, application of pediocin PA-1 or *P. acidilactici* HA-6112 along with HHP led to elimination of the pathogen immediately or 72 h after HHP, indicating the point that there is synergistic effect among pediocin and HHP (Maciel et al., 2017). Similarly, Castro et al. (2018) demonstrated that application of pediocin bacHA-6111-2 (*in situ* and *ex situ*) in combination with HHP was able to effectively control *L. innocua* in fermented meat products.

Furthermore, pediocin can be incorporated to food packaging materials to achieve a possible alternative approach to control *L. monocytogenes* in meats and poultry products. Ming et al. (1997) studied the anti-listeria effect of pediocin addition (at 7.75 μg/cm) into packaging material on turkey breast, ham, and beef. They found that *L. monocytogenes* growth was completely prevented in the samples coated with plastic packaging bags containing pediocin powder during 12 weeks of storage at 4°C. In addition, Woraprayote et al. (2013) observed that *L. monocytogenes* was decreased by 2 log cfu/g in the raw sliced pork that was packed with poly(lactic acid)/sawdust particle biocomposite film incorporated with pediocin PA-1/AcH. Similarly, Santiago-Silva et al. (2009) reported a 2 log cfu/ml reduction on the sliced ham coated with cellulose-based film impregnated with 50% pediocin during 15 days of storage at 12°C. Likewise, it has been observed that application of commercially available pediocin (ALTA^TM^ 2341) in the cellulosic film-forming solution at concentrations of 50% (w/w) resulted in a 1.2 log cfu/g reduction in *L. monocytogenes* population on sliced bologna and hindered biofilm formation on packaging and bologna surfaces (Espitia et al., 2013). Therefore, it seems that pediocin could be a packaging material, as part of the hurdle technology system for the barrier of *L. monocytogenes* in meat and meat products.

As mentioned above, various studies have demonstrated the anti-listeria activity of pediocin in meat and meat products, and this activity is driven by the destruction of bacterial membranes. In fact, the presence of cysteine residues in the structure of the bacteriocin molecule often shows the characteristics of an amphipathic helix, which allows the bacteriocin molecules to begin rearranging the membrane and create pores in the membrane (Nissen-Meyer et al., 1992; Miller et al., 1998; Montville and Chen, 1998). This process is part of membrane lysis, dissipation of PMF and prevention of energy production, inhibition of glucose uptake, and release of cytoplasmic ATP (Montville and Bruno, 1994; Montville and Chen, 1998). It has been indicated that dissipation of PMF and efflux of inorganic phosphate were dependent on the concentration of pediocin and time (Chen and Montville, 1995). As observed in different studies, the effectiveness of the anti-listeria activity of pediocin was affected by various factors such as type of pediocin, type of products, the population of *Listeria*, and initial contamination, time and temperature of storage, and application of complementary inhibitory strategies (Nielsen et al., 1990; Degnan et al., 1992; Luchansky et al., 1992; Chen et al., 2004c; Nieto-Lozano et al., 2006, 2010; Grosulescu et al., 2011; Kingcha et al., 2012; Castro et al., 2018). Furthermore, inactivation of pediocin by protease enzymes, limited diffusion in a solid matrix, binding to food components, and limited effect on Gram-negative bacteria should be considered as the most important challenges regarding the use of pediocin in meat and meat products (Schved et al., 1994; Murray and Richard, 1997). Therefore, most of the studies indicated that pediocin should be used in combination with other inhibitory supplementation methods, especially in the multi-hurdle technology to achieve the highest anti-listeria activity.

## Conclusion

Class IIa bacteriocins are small and cationic proteins with anti-listeria activity. Among these, pediocin presents a conserved N-terminal hydrophobic region in the YGNGV motif and a variable C-terminal hydrophobic or amphiphilic region. Pediocin and pediocin-like bacteriocins show various important technological features such as thermostability and retaining activity at a wide range of pH, which, when accompanied by antibacterial activity against Gram-positive food spoilage and pathogenic bacteria, make them a main class of biopreservatives. This study has revealed that pediocin has promising antimicrobial activity and could be potentially utilized as a natural anti-listeria agent in meat and meat products. The cytoplasmic membrane of bacteria is the target of pediocin, and after contact of pediocin on the surface of the bacterial cytoplasmic membrane, it forms the pores by insertion of the hydrophobic, C-terminal, hairpin-like domain into the membrane. Formation of pores leads to efflux of ions and other cell compounds, release of cytoplasmic ATP, prevention of PMF for energy production, and finally death of cells. Purified pediocin, pediocin in killed cells, and inoculation with pediocin-producing starter cultures are the methods utilized to add this bacteriocin to meat and meat products. Due to the sensitivity of pediocin to proteolytic degradation as well as its binding to food compounds, direct application of pediocin is associated with limitations, particularly in low concentration or during long-term storage at improper temperatures. In this regard, encapsulation of pediocin or its application in combination with other preventative methods could be useful. Generally, it can be stated that pediocin and pediocin-like bacteriocin could be potentially used as part of the hurdle technology along with other preventative strategies or as part of antimicrobial agents incorporated into packaging materials. Furthermore, the application of pediocin with novel technologies such as cold plasma, irradiation, high-intensity pulsed electric field, and HHP against *Listeria* should be investigated in future studies.

## Author Contributions

MY and NK designed this study. EK, NK, and MM wrote the manuscript. MY and AM critically revised the manuscript, and finally, all authors listed have approved it for publication. All authors contributed to the article and approved the submitted version.

## Conflict of Interest

The authors declare that the research was conducted in the absence of any commercial or financial relationships that could be construed as a potential conflict of interest.

## Publisher’s Note

All claims expressed in this article are solely those of the authors and do not necessarily represent those of their affiliated organizations, or those of the publisher, the editors and the reviewers. Any product that may be evaluated in this article, or claim that may be made by its manufacturer, is not guaranteed or endorsed by the publisher.
