# Supplementary material for: Antibacterial Activity of Pediocin and Pediocin-Producing Bacteria Against Listeria monocytogenes in Meat Products
Source: Front Microbiol. 2021 Sep 17;12:709959. doi: 10.3389/fmicb.2021.709959 (PMC8486284; doi:10.3389/fmicb.2021.709959)
Supplement: Supplementary file 1 [file Table_1.docx]

**Table 1.** Antimicrobial activity of cell- free supernatant of *Pediococcus* spp. or isolated pediocin as consequence of various treatments.

| **Pediocin isomoform** | **Producing bacteria** | **Antibacterial Spectrum** | | **pH treatment** | | **Heat treatment** | | **Enzymatic treatment** | | | | | | | | | | **References** |
| --- | --- | --- | --- | --- | --- | --- | --- | --- | --- | --- | --- | --- | --- | --- | --- | --- | --- | --- |
|  |  | G- | G+ | pH 2-10 | pH 4-8 | 100°C/  15 min | 121°C/  15 min | Catalase | Lysozyme | Lipase | Ribonuclease A | Ficin | Trypsin | Chymotrypsin | Proteinase K | Papain | Pepsin |  |
| Z-1 | *P. Pentosaceus* Z-1 | + | + | + | + | + | ND | ND | ND | + | ND | ND | - | ND | - | - | - | (Yu et al., 2020) |
| GS4 | *P. pentosaceus* GS4 | + | + | - | ± | - | ND | ND | + | ND | ND | ND | ND | ND | ND | ND | - | (Ghosh et al., 2019) |
| KJBC11 | *P. pentosaceus* KJBC11 | + | + | ± | + | + | - | + | ND | + | ND | ND | - | ND | - | ND | ND | (Sadishkumar and Jeevaratnam, 2018) |
| CFS | *P. pentosaceus* KID7 | + | + | ND | ND | ND | ND | ND | ND | ND | ND | ND | ND | ND | ND | ND | ND | (Damodharan et al., 2015) |
| SB83 | *P. pentosaceus* SB83 | - | + | + | + | + | - | + | ND | + | ND | ND | - | - | - | - | - | (Borges et al., 2014) |
| NCIM 2292 | *P. acidilactici* NCIM 2292 | - | + | + | + | + | + | + | ND | + | ND | ND | - | - | - | - | - | (Mandal et al., 2014) |
| AcH/PA-1 | *P. pentosaceus* OZF | ND | + | + | + | + | + | + | + | + | + | - | - | - | - | ND | ND | (Osmanagaoglu et al., 2011) |
| ST44AM | *P. pentosaceus*  ST44AM. | ± | ± | + | + | + | ND | + | ND | + | ND | ND | - | - | - | - | - | (Todorov and Dicks, 2009) |
| SM-1 | *P. pentosaceus* Mees | - | + | + | + | + | + | ND | ND | ND | ND | ND | - | - | - | - | - | (Anastasiadou et al., 2008a) |
| pK23-2 | *P. pentosaceus* K23-2 | - | + | ± | + | + | + | + | ND | ND | ND | ND | + | ND | - | ND | - | (Shin et al., 2008) |
| SA-1 | *P. acidilactici*  NRRL B5627 | - | + | + | + | + | + | ND | ND | ND | ND | ND | + | + | - | + | + | (Anastasiadou et al., 2008b) |
| A | *P. pentosaceus* ATCC25745 | ND | + | ND | ND | + | ND | ND | ND | ND | ND | ND | ND | ND | + | ND | ND | (Diep et al., 2006) |
| **Table 1.** Continued**.** | |  |  |  |  |  |  |  |  |  |  |  |  |  |  |  |  |  |
| **Pediocin isomoform** | **Producing bacteria** | **Antibacterial Spectrum** | | **pH treatment** | | **Heat treatment** | | **Enzymatic treatment** | | | | | | | | | | **References** |
|  |  | G- | G+ | pH 2-10 | pH 4-8 | 100°C/  15 min | 121°C/  15 min | Catalase | Lysozyme | Lipase | Ribonuclease A | Ficin | Trypsin | Chymotrypsin | Proteinase K | Papain | Pepsin |  |
| ST18 | *P. pentosaceus* ST18 | - | ± | + | + | + | + | ND | ND | ND | ND | ND | ND | ND | ND | ND | ND | (Todorov and Dicks, 2005) |
| ACCEL | *P. pentosaceus* ACCEL | - | + | + | + | + | ± | ND | ND | ND | ND | ND | ND | - | ND | ND | - | (Wu et al., 2004) |
| S | *P. pentosaceus* S | ± | ± | - | + | + | - | ND | ND | ND | ND | ND | ND | - | ND | ND | - | (Yin et al., 2003) |
| L | *P. pentosaceus* L | ± | ± | - | + | + | - | ND | ND | ND | ND | ND | ND | - | ND | ND | - | (Yin et al., 2003) |
| F | *P. acidilactici* F | ND | + | + | + | + | + | + | + | + | + | - | - | - | - | - | ND | (Osmanagaoğlu et al., 1998) |
| PD-1 | *P. damnosus* NCFB 1832 | ND | + | + | + | + | ND | ND | ND | ND | ND | ND | + | + | - | + | + | (Green et al., 1997) |
| ATO34 and  ATO77 | *P. parvulus* ATO34 and  ATO77 | - | + | - | + | + | ND | ND | ND | ND | ND | ND | - | - | - | - | - | (Bennik et al., 1997) |
| N5p | *P. pentosaceus* N5p | ND | ND | - | + | + | + | ND | ND | ND | ND | ND | ND | ND | ND | ND | ND | (de Saad et al., 1995) |
| A | *P. pentosaceus* FBB61 | - | + | ND | ND | + | ND | ND | ND | ND | ND | ND | + | ND | + | ND | ND | (Piva and Headon, 1994) |
| SJ-1 | *P. acidilactici* SJ-1, | - | + | - | + | + | + | ND | ND | ND | ND | ND | - | - | - | - | ND | (Schved et al., 1993) |
| AcH | *P. acidilactici* H | ND | + | ND | ND | ND | ND | ND | ND | ND | ND | ND | - | ND | ND | ND | ND | (Bhunia et al., 1991) |
| PA-1 | *P.acidilactici* PAC 1.0 | ND | + | + | + | + | + | ND | + | + | ND | ND | ND | - | ND | - | - | (Gonzalez and Kunka, 1987) |

Antimicrobial activity of Pediocin or cell-free supernatants of *Pediococcus* ssp.; (+): activity, (−): absence of any activity or ND: not determined.
